# Improvement of the Texture of Yogurt by Use of Exopolysaccharide Producing Lactic Acid Bacteria

**DOI:** 10.1155/2016/7945675

**Published:** 2016-05-15

**Authors:** Xue Han, Zhe Yang, Xueping Jing, Peng Yu, Yingchun Zhang, Huaxi Yi, Lanwei Zhang

**Affiliations:** ^1^School of Chemical Engineering & Technology, Harbin Institute of Technology, Harbin 150090, China; ^2^Dairy Research Institute, Bright Dairy & Food Co., Ltd., Building 2, No. 1518, West Jiangchang Road, Shanghai 200436, China

## Abstract

19* Streptococcus thermophilus* with high exopolysaccharide production were isolated from traditional Chinese fermented dairy products. The exopolysaccharide and viscosity of milk fermented by these 19 isolates were assayed. The strains of* Streptococcus thermophilus* zlw TM11 were selected because its fermented milk had the highest exopolysaccharide content (380 mg/L) and viscosity (7716 mpa/s). Then* Streptococcus thermophilus* zlw TM11 was combined with* Lactobacillus delbrueckii* subsp.* bulgaricus* 3 4.5 and the combination was named SH-1. The quality of the yogurt fermented by SH-1 and two commercial starter cultures (YO-MIX 465, YF-L711) were compared. It was shown that the exopolysaccharide content of yogurt fermented by SH-1 was similar to that of yogurt fermented by YF-L711 and significantly higher than YO-MIX 465 (*p* < 0.05). In addition, the yogurt fermented by SH-1 had the lowest syneresis (8.5%) and better texture and sensory than the samples fermented by YO-MIX 465 and YF-L711. It manifested that the selected higher exopolysaccharide production starter SH-1 could be used as yogurt starter and reduce the amount of adding stabilizer, which can compare with the imported commercial starter culture.

## 1. Introduction

Yogurt has been an integral part of everyday diet for centuries, rising as the second most popular snack among children in the world [1]. Textural properties of yogurt, such as viscosity [2], smoothness and thickness [3], and structural resistance to stress [4], are important attributes to determine its consumer acceptance, and these attributes nowadays are accompanied with certain health benefits [5]. Many methods have been used to improve the quality of the yogurt, such as increasing the solids in milk (adding fat, proteins, or sugars such as sucrose and fructose), addition of stabilizers (pectin, starch, alginate, and gelatin) [6, 7]. However, these approaches did not satisfy the consumers demand for products with as few food additives as possible. Exopolysaccharide (EPS) produced by LAB with GRAS (generally recognized as safe) status is an important source of natural alternatives. Recently, EPS produced by LAB have gained considerable attention in the fermented dairy industry because of their potential application as viscosifiers, texturizers, and emulsifying agents [8]. It had been reported that EPS produced by yogurt starter cultures could affect the texture of yogurt and improve sensory characteristics such as mouthful, shininess, clean cut, ropiness, and creaminess [5]. Furthermore, the yogurt cultures producing EPS may decrease the extent of syneresis (the lower whey separation) [9]. Syneresis is considered as a major defect in yogurt and connected with extensive rearrangements of the gel network [10]. Presently, EPS plays a major role in the production of fermented dairy products in Northern Europe, Eastern Europe, and Asia [6]. However, the EPS of LAB are in a great variety, which depends on the type of LAB strains, culture conditions, and medium composition [9, 11]. It is significant to select the strains with higher EPS production.

*Streptococcus thermophilus* and* Lactobacillus delbrueckii* subsp.* bulgaricus* are the mainly strains used in the yogurt starter culture and used for yogurt production. Unfortunately, some of strains of* Lactobacillus delbrueckii* subsp.* bulgaricus* and* Streptococcus thermophilus* did not produce EPS or produce only low yields of EPS, which may affect the end products quality [12–14]. Presently, most of the yogurt starter cultures were imported from Denmark Danisco or Chr Hansen in China. And Chinese own authority starter culture was scarcity.

In China, there are many traditional fermented dairy products. Therefore, screening LAB from natural sources has been one of the powerful means to obtain strains for the food industry. We have already screened LAB from Chinese traditional fermented dairy products and some properties have been characterized in our previous work [15, 16]. In this study, it was mainly focused on the exopolysaccharide production strains by* Streptococcus thermophilus*. The aim of this work was to select the starter culture with higher exopolysaccharide production to improve the yogurt quality and develop our own authority yogurt starter culture. The selected higher exopolysaccharide production starter will reduce the amount of additives addition and can be comparable with the imported starter culture.

## 2. Materials and Methods

### 2.1. Materials

The commercial yogurt starters, YO-MIX 465 and YF-L711, were obtained from Danisco and Chr. Hansen (Denmark). The whole cow raw milk was purchased from supermarket (Harbin, China). Skim milk was purchased from Nestle Company, Heilongjiang, China. All the chemicals used in this paper were of analytical grade.

### 2.2. Exopolysaccharide Producing Strains Selection

19* Streptococcus thermophilus* were screened from traditional Chinese fermented dairy products. The above 19 isolates were inoculated into the sterilized (121°C, 15 min) reconstituted skim milk (12%), respectively. The inoculate rate was 2% and incubated at 42°C until pH attained to 4.7. The fermented samples were cooled at 4°C for 12 h. Then the content of exopolysaccharide and viscosity in fermented milks were detected. The* Streptococcus thermophilus*, which produced higher exopolysaccharide and had higher viscosity, was selected. The pH of each sample was determined directly using a digital pH meter (PB-10, Sartorius, Germany) calibrated with standard buffer solutions. Three replicates were performed.

### 2.3. Yogurt Starter Culture

The selected* Streptococcus thermophilus* with higher exopolysaccharide production was combined with* Lactobacillus delbrueckii* subsp.* bulgaricus* 3 4.5, which was screened from Chinese traditional fermented dairy products and identified by Biolog identification system and analysis of 16S rDNA gene sequence. The direct vat starter (DVS) SH-1 which was composed by these two stains (the fermented characteristic about the combined starter culture was reported in our previous work [16]) was used to ferment the cow milk. The fermented cow milk produced by the commercial yogurt starter, YO-MIX 465 and YF-L711, was used as contrasts. Then the exopolysaccharide content, texture, syneresis, and sensory of fermented cow milk were evaluated.

### 2.4. Yogurts Preparation

Raw fresh cow milk consisted of 3.14% protein, 3% fat, lactose 4.92%, and 11.81% total solids, was analyzed by MilkoScan FT1 (Denmark), and then preheated to 65°C and homogenized. The homogenized milk was pasteurized at 65°C for 30 minutes and cooled to 42°C and portioned to three equal batches. The batches were inoculated with about 3 mg/L starter cultures (YO-MIX 465, YF-L711, and SH-1) that were determined by the initial viable counts of yogurt. The initial viable counts of each batches were about 10^7^ CFU/mL measured by spread plate count method and incubated at 42°C until the pH reached 4.6 (about 4 to 4.5 h). Then yogurt samples were put into the refrigerating chamber (4°C) for 12 h to detect the EPS production, appearance viscosity, texture, syneresis, and sensory of yogurts. Each test has three replications.

### 2.5. Exopolysaccharides Detection

EPS was isolated from the fermented sample, according to a modified method of Lin and Chien [8]. The fermented sample was added with an equal volume of trichloroacetic acid (40%); the precipitated protein and bacteria were removed by centrifugation (2200 g for 35 min at 4°C). The supernatant was then mixed with an equal volume of ethanol, stored at 4°C for 24 h, and centrifuged as described above to collect the precipitated EPS. The EPS was then dissolved in water. The total neutral glycoside content was determined by the phenol-sulphuric acid method [17].

### 2.6. Viscosity Measurement

Viscosity measurement was carried out at 4 ± 1°C by Brookfield Programmable DV-E Viscometer (Brookfield Engineering Laboratories, Inc., Middleboro, MA, USA) equipped with S63 spindle at 20 rpm.

### 2.7. Texture Analysis

The texture of yogurt was determined by penetration measurements (TA.XT plus, Texture Analyzer, Stable Micro Systems Ltd., England). The instrument was adjusted to the following conditions: cylindrical probe, probe diameter: 35 mm; penetration speed: 1.0 mm/s; penetration distance, 20 mm into surface. The software used was Exponent (Stable Micro Systems, 2006, version 5.0). 100 mL of yogurt sample was analyzed in each cup. Four parameters were evaluated, the firmness (g) (maximum force, i.e., exerted on the sample), defined as the force necessary to attain a given deformation; the cohesiveness (g/s) (adhesive force), defined as forces of internal bonds, which keep the product complete; the viscosity (g); and the adhesiveness (g/s) (total negative area); the work is necessary for overcoming the force of attraction between the area of foodstuff and other solids coming to contact with them.

### 2.8. Syneresis Measurement

Syneresis index of different yogurt samples was determined according to the methodology proposed by Farnsworth et al. [18] with modifications. Yogurt (20 g) was prepared in centrifuge cups and centrifuged at 350 ×g (model K-24; Sigma Laborzentrifugen GmbH, Osterode am Harz, Germany) for 10 min at 4°C. The clear supernatant was collected and weighed and syneresis was calculated according to the following equation [18, 19]:

(1) Syneresis%=weight  of  supernatant  gweight  of  yogurt  sample  g×100%.

### 2.9. Sensory Evaluation

Twenty trained panelists (fourteen women and six men, aged 22–45) were asked to evaluate the sensory attributes of yogurt. The ratings were presented on a 9-point hedonic scale ranging from 9 (“like extremely”) to 1 (“dislike extremely”). Yogurt sensory parameters were evaluated by thickness, smoothness, fermented odor, finished flavor, and taste quality. To minimize bias, all groups were three digits coded. The yogurts were served to panelists after the cooling process. Result was given on averages of the three trials for each type of yogurt [20].

### 2.10. Statistical Analysis

Statistical analyses were performed using SPSS 14.0 software (SPSS Inc.; Chicago, IL, USA). Significant differences among treatments were tested by ANOVA followed by Tukey's test with a level of significance at *α* = 0.05. Data were expressed as mean values ± standard deviation (S.D.). For analyzing the relationship of exopolysaccharide production and viscosity of fermented milk, the correlate bivariate analysis was used to determine the correlation coefficient. All experiments were performed in duplicate and repeated three times.

## 3. Results and Discussion

### 3.1. Exopolysaccharide Production Strains Selection

The exopolysaccharide production and the viscosity of fermented cow milk produced by the 19* Streptococcus thermophilus* isolated from traditional Chinese fermented dairy products were assayed. In Table 1, it showed that different strains had different exopolysaccharide production and viscosity. For the fermented milk produced by* Streptococcus thermophilus* zlw TM11, the exopolysaccharide and viscosity were significantly higher than that of the other strains (*p* < 0.05). The relationship of exopolysaccharide production and viscosity of fermented milk was analyzed by SPSS software correlate bivariate analysis. The results showed that the correlation coefficient between exopolysaccharide production and viscosity was 0.841 (*p* < 0.01). It suggested the strains producing higher exopolysaccharide might contribute to the higher viscosity of fermented milk. Guzel-Seydim et al. [9] reported that exopolysaccharide filaments attached mucous bacteria to the protein matrix and thus caused more viscous-like behavior. Many researchers also reported that yogurts made with polysaccharide producing cultures had higher viscosity values than the yogurts made with none of the polysaccharide production cultures [21, 22]. Therefore, the higher exopolysaccharide producing strains of* Streptococcus thermophilus* zlw TM11 were selected to combine with* Lactobacillus delbrueckii* subsp.* bulgaricus* 3 4.5. The combination was named SH-1 and was used as starter to make yogurt.

### 3.2. Content of Exopolysaccharides in Yogurt

**Figure 1:**
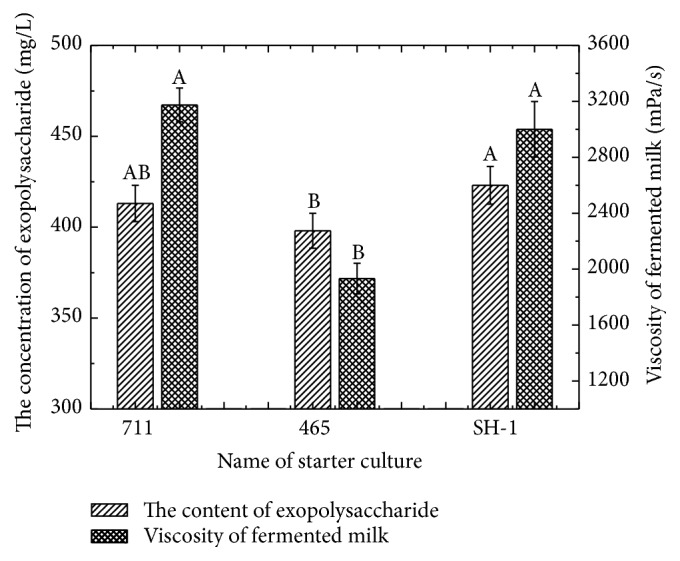
The concentration of exopolysaccharide in yogurt fermented by different starter culture. Mean values (*n* = 3), 465 was the starter of YO-MIX 465, 711 was the starter of YF-L711, and SH-1 was the screened starter. A, B, and treatments with different letters are different at *p* < 0.05. Error bars indicate standard deviation.

The exopolysaccharide content in each yogurt samples was showed in Figure 1. For yogurt sample produced by SH-1, the contents of exopolysaccharide and viscosity were significantly higher than that of sample produced by YO-MIX 465 (*p* < 0.05) and had no significant difference with YO-MIX 711 (*p* > 0.05). Starter YO-MIX 711 had the higher exopolysaccharide production and also had higher viscosity in fermented milk compared with YO-MIX 465, although there was no significant difference for the fermented milk viscosity (*p* > 0.05). It was verified in the result of Table 1 that the strains with higher exopolysaccharide production had the higher viscosity. And it also indicated that the exopolysaccharide production was different from the types of starter culture [11].

### 3.3. Texture Properties

**Figure 2:**
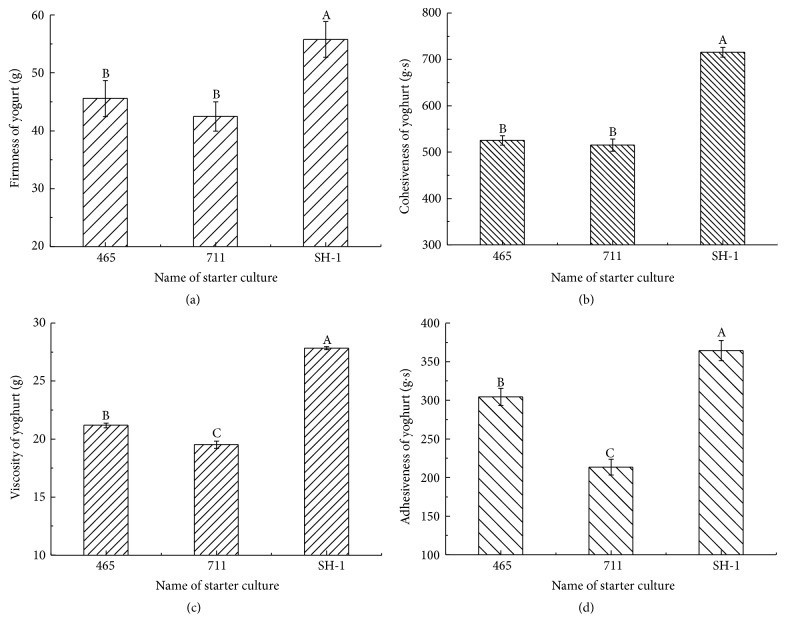
The texture of different starter cultures fermented yogurt. (a) The firmness of yogurt; (b) the cohesiveness of yogurt; (c) the viscosity of yogurt; (d) the adhesiveness of yogurt. 465 was the starter of YO-MIX 465, 711 was the starter of YF-L711, and SH-1 was the screened starter. A, B, and C: treatments with different letters are different at *p* < 0.05. Error bars indicate standard deviation.

The textures properties of the yogurts fermented by different starter cultures were showed in Figure 2. The concentration of exopolysaccharide of SH-1 was the highest (423.05 mg/L), corresponding to the highest firmness (55.77 g), highest cohesiveness (715.31 g·s), highest viscosity (27.83 g), and highest adhesiveness (364.30 g·s), respectively. The YF-L711 has the lowest firmness (36.70 g), cohesiveness (457.86 g·s), viscosity (18.63 g), and adhesiveness (212.10 g·s). These factors are significantly lower than starter SH-1 (*p* < 0.05). For YF-L711, the firmness (36.70 g) and cohesiveness had no significantly different with YO-MIX 465 (*p* > 0.05). While viscosity (18.63 g) and adhesiveness were significantly lower than YO-MIX 465 fermented yogurt (*p* < 0.05). The content of exopolysaccharide in YF-L711 fermented yogurt was a little higher than that of YO-MIX 465 fermented yogurt, though no significant difference (*p* > 0.05) was observed. It was inferred that a little increase of exopolysaccharide might have great effect on viscosity and adhesiveness.

Many researchers reported that the exopolysaccharide could improve the texture of yogurt, because exopolysaccharide produced by LAB interacts with the free water in the gel-like structure [9, 23, 24]. In this study, the yogurt fermented by SH-1 had the highest content of exopolysaccharide and the best texture than the yogurt fermented by the other starter cultures. It suggested that the selected starter SH-1 could reduce some stabilizer addition and replace the imported commercial starters. Similar results were found by Patel et al. [22]. They reported that exopolysaccharide could improve the quality of yogurt, while having no effect on flavor of yogurt. Therefore, it could replace some stabilizer. However, the concentration of polysaccharide and the texture of yogurt had no linear correlation. It was assumed that the texture of yogurt could be affected by starter types and the structure of exopolysaccharide [25].

### 3.4. Syneresis of Yogurt

**Figure 3:**
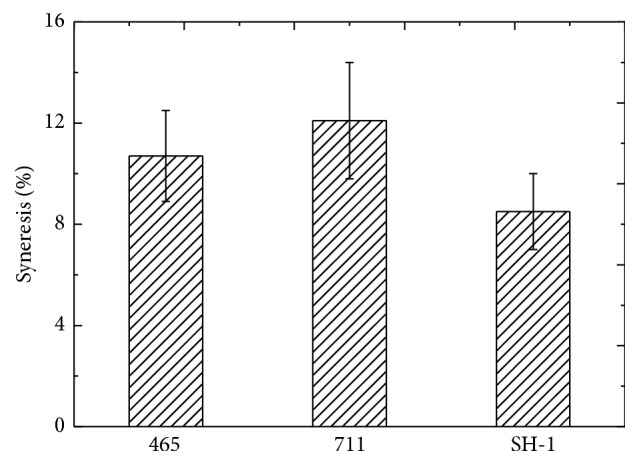
The syneresis of yogurt fermented by different starter culture. 465 was the starter of YO-MIX 465, 711 was the starter of YF-L711, and SH-1 was the screened starter. There are no significant differences at *p* > 0.05. Error bars indicate standard deviation.

The syneresis of samples fermented by different starters was showed in Figure 3. There were no statistical differences for these three samples (*p* > 0.05). Among them, starter SH-1 had the highest exopolysaccharide content (423 mg/L), corresponding to the lowest whey separation (8.51%). For commercial starters YO-MIX 465 and YF-L711, the syneresis was 10.70% and 12.12%, respectively. It seemed that the higher content of exopolysaccharide would contribute to the lower whey separation. Similar results had also been reported by other studies [2]. This might be due to the high water-binding capacity of EPS as well as modifications of yogurt microstructure by EPS cultures [24, 26]. Thus, the yogurts made from EPS producing starters showed better textural characteristics.

### 3.5. Sensory Evaluation

**Figure 4:**
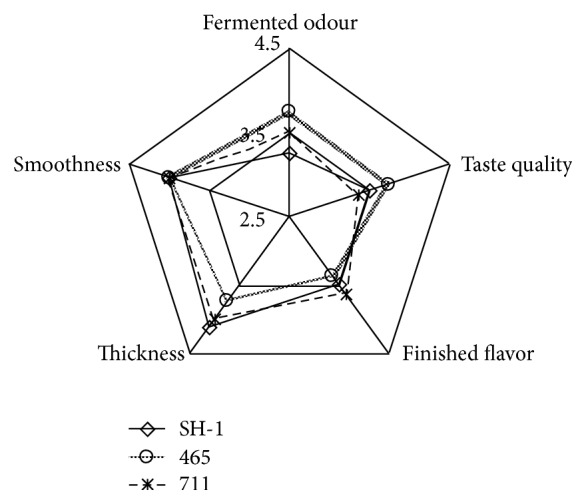
Sensory value for different starter fermented milk. 465 was the starter of YO-MIX 465, 711 was the starter of YF-L711, and SH-1 was the screened starter.

The scores for sensory characteristics of the yogurt samples were presented in Figure 4. For thickness, the selected starter culture SH-1 was better than YO-MIX 465 and similar to YF-L 711. For smoothness, the starter SH-1 was similar to YO-MIX 465 and YF-L 711. For taste quality, the scores of selected starter SH-1 were higher than YF-L 711 but lower than YO-MIX 465. For the finished flavor, selected starter SH-1 has no significant difference from YF-L 711 and YO-MIX 465 (*p* > 0.05). As regards to the fermented odor, the starter SH-1 was no better than YO-MIX 465 and YF-L 711. The results of sensory evaluation indicated that the selected starter SH-1 had the potential to replace the imported commercial starter.

## 4. Conclusion

The starter culture type affected the qualities of yogurt samples. The selected starter SH-1 had the similar content of exopolysaccharides to the commercial starter YF-L 711 and significantly higher than YO-MIX 465. The higher exopolysaccharides could provide better texture of yogurt and lower whey separation. On the other hand, the sensory evaluation of yogurt using selected culture SH-1 was better than or similar to the other commercial starter cultures, except for the fermented odor which was a little lower than the other two commercial starters. These implied that our own authority starter SH-1 could produce the yogurt with similar or better quality compared with the commercial starters.

## Figures and Tables

**Table 1:** The content of exopolysaccharide and viscosity for different *Streptococcus thermophilus* fermented milk (values are means ± SD for *n* = 3).

Strains	EPS (mg/L)	Viscosity (mPa/s)
zlwTM11	384 ± 22^a^	7716 ± 608.80^A^
zlwB9-3	255 ± 43^b^	5960 ± 1716.77^B^
zlwQ	175 ± 54^c^	4360 ± 685.85^C^
zlwA1	155 ± 2^cd^	4240 ± 58.89^C^
zlwA2M17	141 ± 29^cde^	4198 ± 340.33^C^
zlw17YA	128 ± 5^de^	3450 ± 42^CDE^
zlwCH9.9 4.0	127 ± 7^de^	3804 ± 27.50^CD^
zlw3	121 ± 5^de^	3562 ± 101.17^CD^
zlwLBH	120 ± 5^de^	2920 ± 203.15^DEF^
zlw1703Ca	115 ± 3^de^	2870 ± 84.92^DEF^
zlwCH9-94.5	101 ± 57^dfg^	2210 ± 206.14^F^
zlw94.3	99 ± 2^efg^	2726 ± 97.73^EF^
zlwSP1.1	93 ± 10^fgh^	2726 ± 383.26^EF^
zlw1703F	85 ± 34^fgh^	2722 ± 1084.76^EF^
zlw1	73 ± 6^ghi^	1206 ± 64.90^G^
zlwDV	56 ± 12^ghi^	2194 ± 272.35^F^
zlw1703D	54 ± 16^hi^	2556 ± 33.41^EF^
zlwSH94.3	47 ± 13^i^	2303 ± 121.39^F^
zlwSH94.5	45 ± 7^i^	1092 ± 43.27^G^

a, b, c, d, e, f, g, h, and i: values with different letters within the same column differ significantly (*p* < 0.05) for exopolysaccharide. A, B, C, D, E, F, and G: values with different letters within the same column differ significantly (*p* < 0.05) for viscosity.
